# Fixed Buffer Zone Legislation: A Proportionate Response to Demonstrations Outside Abortion Clinics in England and Wales?

**DOI:** 10.1093/medlaw/fwac019

**Published:** 2022-06-24

**Authors:** Emily Ottley

**Affiliations:** Dickson Poon School of Law, King’s College London, UK

**Keywords:** Access to abortion, Buffer zones, European Convention on Human Rights, General clinic protest, Offence principle, Proportionality

## Abstract

There is concern that the recent increase in demonstrations outside abortion clinics in England and Wales may have a detrimental impact on clinic-users’ access to abortion services. Parliament could respond to this concern by passing legislation that implements fixed buffer zones around all clinics providing abortion services in England and Wales. This would make it an offence to engage in prohibited behaviour (as defined by the legislation) within a specified area around abortion clinics. Such legislation may be challenged, however, on the basis that it interferes with the rights afforded to demonstrators by Articles 9, 10, and 11 of the European Convention on Human Rights (ECHR). This article examines the proportionality of fixed buffer zone legislation, which has not yet been considered by the European Court of Human Rights nor the UK Supreme Court. Two relationships are considered: first, the relationship between the aims of the measures and the means to achieve those aims; second, the relationship between the competing interests of demonstrators opposing abortion and clinic-users seeking an abortion. This article shows that fixed buffer zone legislation can be proportionate. Consequently, the ECHR is no impediment to the enactment of fixed buffer zone legislation in England and Wales.

## I. INTRODUCTION

In 2020, 77% of abortions funded by the National Health Service (NHS) in England and Wales were performed in independent sector clinics as opposed to NHS hospitals.^1^ Increasingly, clinic-users encounter demonstrations by pro-life advocates who oppose abortion.^2^ Cohen and Connor call this ‘general clinic protest’.^3^ The activities undertaken as part of the demonstrations outside abortion clinics are wide-ranging and it is not possible to produce an exhaustive list. Nevertheless, Sarah Champion MP provides a useful summary of typical protest activities:

… the display of graphic images of dismembered foetuses, large marches that gather outside the clinic, filming women and staff members, following women down the street, sprinkling sites with holy water and handing out leaflets that tell women, falsely, that abortion causes breast cancer, suicidal intentions and can lead to child abuse. Recently, groups have been handing out advertisements for dangerous and unproven medication to reverse an abortion.^4^

Obstructing clinic entrances, shouting at clinic-users (often addressed as ‘mum’ or a ‘murderer’), grabbing clinic-users, and praying are further examples of typical protest activities.^5^ It is the presence of demonstrators outside clinics that is the main focus of this article, however, rather than any particular one of these activities. Further, this article concentrates on the effects of demonstrators on clinic-users rather than clinic staff and/or the local community.

These demonstrations are striking because they seem to be intended to affect the decisions of individual clinic-users to have an abortion, rather than to oppose abortion law or policy in any effective way.^6^ This is apparent from the location of the demonstrations, which occur outside abortion clinics rather than the Houses of Parliament. Indeed, pro-life advocates use ‘general clinic protest’ to both directly and indirectly affect a clinic-user’s decision to terminate the pregnancy. The demonstrators’ aim is to dissuade clinic-users from having an abortion with their actions and/or to deter clinic-users from entering the clinic with their presence.^7^ Doctors and other clinic staff may also be targeted by demonstrators in an attempt to restrict/limit the availability of safe legal abortion so that it is not an option for clinic-users.^8^

Little is known, however, about whether demonstrations outside abortion clinics actually dissuade or deter clinic-users from having an abortion.^9^ Further, it is not obvious how such research could be undertaken because interviewing clinic-users at a clinic may not include those who were deterred/dissuaded from entering the clinic.^10^ Nevertheless, deterrence and dissuasion is a real possibility.^11^ There is also anecdotal evidence that some clinic-users in England and Wales are rebooking (delaying) their appointments due to demonstrations outside abortion clinics.^12^ Even if the demonstrations do not change a clinic-user’s decision to terminate the pregnancy on that day, the presence of demonstrators outside abortion clinics patently makes implementing that decision more difficult.

The view adopted in this article is that abortion is healthcare. This is in line with that taken by the Royal College of Obstetricians and Gynaecologists.^13^ Therefore, a clinic-user’s ‘refusal’ to have an abortion must be voluntary.^14^ A clinic-user’s ‘refusal’ to have an abortion will not be voluntary where she has been coerced or unduly influenced by demonstrators.^15^ In contrast, demonstrators may not see abortion as healthcare. Nevertheless, abortions can be performed lawfully in the circumstances prescribed by section 1 of the Abortion Act 1967. Therefore, a clinic-user must be free to consult a doctor and to access abortion in these circumstances.

One response to ‘general clinic protest’ would be for the UK Parliament to pass legislation that implements fixed buffer zones around all clinics providing abortion services in England and Wales. This would make it an offence to engage in prohibited behaviour (as defined by the legislation) within a specified area around abortion clinics. Such legislation already exists in Australia.^16^ Some form of fixed buffer zone legislation also exists in six Canadian provinces, though this does not protect all clinics in the province *automatically* (except in Quebec).^17^ Both Canadian and Australian legislation has survived constitutional challenge on the grounds of freedom of expression and freedom of political communication respectively.^18^ In the UK, a Bill that would allow a fixed buffer zone to be implemented upon the request of an abortion clinic was passed by the Northern Ireland Assembly on 24 March 2022.^19^ Further, there have been recent attempts to pass fixed buffer zone legislation in the UK Parliament. The Demonstrations (Abortion Clinics) Bill (the Bill) failed to complete its passage through Parliament before the end of the session and an amendment to the Police, Crime, Sentencing and Courts Bill (the amendment) was not voted on.^20^

If fixed buffer zone legislation is eventually passed by the UK Parliament, it may be challenged on the basis that it interferes with the rights of demonstrators afforded to them by the European Convention on Human Rights (ECHR). The right to freedom of expression under Article 10 ECHR and the right to freedom of assembly under Article 11 ECHR are the most obvious grounds on which to challenge fixed buffer zone legislation.^21^ The Commission held that Article 10 ECHR was engaged in *Van Den Dungen v The Netherlands*, where ‘the applicant was prohibited [by a court injunction] from addressing people and handing out leaflets in the direct vicinity of the abortion clinic’.^22^ Similarly, there was no question in the Court of Appeal that the claimants’ rights under Article 10 ECHR were engaged in *Dulgheriu and another v Ealing London Borough Council*, where a Public Space Protection Order (PSPO) prevented all abortion-related protest within 100m of an abortion clinic.^23^ The claimants’ rights under Article 11 ECHR were also engaged in *Dulgheriu*,^24^ but the applicant in *Van Den Dungen* could not rely on Article 11 ECHR because he had acted alone (meaning there was no assembly).

It is less clear whether the right to freedom of thought, conscience and religion under Article 9 ECHR would be engaged, however.^25^ A demonstrator may be motivated by religious or non-religious convictions about abortion,^26^ but there is some doubt whether the demonstrations constitute a *manifestation* of these beliefs.^27^ In *Van Den Dungen*, the Commission found that the applicant’s activities did not constitute a manifestation of his belief because they ‘were primarily aimed at persuading women not to have an abortion’.^28^ Similarly, the Commission found that distributing leaflets to soldiers urging them to refuse to go to war in Northern Ireland was not a manifestation of the applicant’s pacifist beliefs.^29^ In *Van Schijndel v The Netherlands*, where the applicants were charged with breach of the peace for entering an abortion clinic in order to pray in a corridor, the Commission cited its earlier decision in *Van Den Dungen*.^30^ However, the Commission declined to consider the issue of manifestation in any great detail and accepted (for the sake of argument) that the applicants’ activities could be regarded as a manifestation of their religious beliefs.^31^ The same approach was taken by Turner J when *Dulgheriu* was at the High Court.^32^ The Court of Appeal then offered no useful guidance on this point, as it ‘did not need to resolve’ this question in order to deal with the grounds for appeal.^33^

Despite the apparent conflict between *Van Den Dungen* and *Van Schijndel*, the two cases might be distinguished on the basis of the activities undertaken by the applicants. The applicants in *Van Schijndel* were praying and it is perhaps more obvious that prayer is ‘intimately linked to the religion or belief’ than distributing leaflets and displaying graphic images (as in *Van Den Dungen*).^34^ Indeed, some demonstrators distinguish prayer-vigils from (what they consider to be) protest.^35^ Further, the respondents in *Dulgheriu* were willing to accept that ‘vigils and other acts of prayer’ were protected by Article 9 ECHR, but not any of the other activities undertaken by the applicants.^36^ These other activities may be described as ‘acts … which do not directly express the belief concerned or which are only remotely connected to a precept of faith’.^37^ Rather, they are undertaken by demonstrators to dissuade a clinic-user from having an abortion.^38^ However, it is surprising that displaying images of Christ was not regarded as a manifestation of the applicant’s religious belief in *Van Den Dungen*.^39^ Therefore, only *some* activities undertaken by demonstrators outside abortion clinics (those relating to prayer) are likely to be protected by Article 9 ECHR.

This article examines the proportionality of fixed buffer zone legislation, which is both an important and contentious aspect of justifying the interference under the qualifications to Articles 9, 10, and 11 ECHR.^40^ To date, neither the European Court of Human Rights (ECtHR) nor the UK Supreme Court (UKSC) have considered this issue directly. In order to assess the proportionality of the interference, two relationships will be considered. The relationship between the aims of the measures and the means to achieve those aims will be considered first. The means include both the national aspect of the buffer zone legislation and the nature of the prohibition (as defined by the legislation). The relationship between the competing interests of demonstrators opposing abortion and clinic-users seeking an abortion will be considered second.

## II. THE RELATIONSHIP BETWEEN THE AIMS AND THE MEANS: NATIONAL LEGISLATION

In his statement on the outcome of the Home Office’s Abortion Clinic Protest Review in 2018, Sajid Javid (the then Secretary of State for the Home Department) gave three reasons why ‘introducing national buffer zones would not be a proportional response’.^41^ The first of these was that demonstrations take place outside only a small number of clinics.^42^ However, the number of demonstrations is increasing.^43^ In any event, it is not obvious that buffer zones imposed around abortion clinics where no demonstrations have previously taken place interfere with the rights of pro-life advocates because no demonstrations were taking place there anyway.

The second reason provided by Javid was that the majority of activities that occur outside abortion clinics are ‘passive’.^44^ It is not clear exactly what Javid meant by ‘passive’, but he does offer some examples: ‘praying, displaying banners and handing out leaflets’.^45^ Javid contrasts these ‘passive’ activities with activities he considers to be more ‘aggressive’, such as ‘handing out model foetuses, displaying graphic images, following people, blocking their paths and … assaulting them’.^46^ It is true that demonstrations outside abortion clinics in England and Wales are not usually violent. Indeed, the typical protest activities outlined at the beginning of this article did not include the use of physical force to injure someone or to damage something.

Nevertheless, it is questionable whether there is such a stark difference between displaying (i) banners (passive) and (ii) graphic images (aggressive) or handing out (i) leaflets (passive) and (ii) model foetuses (aggressive). Presumably, Javid is making the point that the ‘passive’ activities are somehow not as bad as the ‘aggressive’ activities. However, the impact of ‘passive’ activities on clinic-users may be just as significant as the more ‘aggressive’ activities. As the Australian High Court noted in *Clubb v Edwards*, ‘[s]ilent but reproachful observance of persons accessing a clinic for the purpose of terminating a pregnancy may be as effective, as a means of deterring them from doing so, as more boisterous demonstrations’.^47^ The Australian High Court further observed that the line between ‘peaceful protest’ and ‘virulent or even violent expression’ is ‘easily and quickly crossed’.^48^

The third reason provided by Javid was that (civil and criminal) ‘legislation already exists to restrict protest activities that cause harm to others’.^49^ Javid’s use of the word ‘harm’ is interesting and may have been carefully considered. This is because the Harm Principle has been influential in Western liberal democracies, such as England and Wales, as a limit on what conduct the State can legitimately criminalise. Mill’s is the most famous formulation of this principle: ‘the only purpose for which power can rightly be exercised over any member of a civilised community against his will is to prevent harm to others’.^50^ For fixed buffer zone legislation to be legitimate according to this principle, it must prevent harm to others. An important question, then, is whether demonstrations outside abortion clinics in England and Wales harm clinic-users. If not, fixed buffer zone legislation cannot be said to *prevent* harm.

The starting point is to consider the adverse consequences of the demonstrations for clinic-users. The adverse consequences for a clinic-user who is prevented from having an abortion are well known. First, a lack of choice in reproductive decisions has been linked to mental health problems.^51^ Second, a clinic-user will have to endure the significant physical burdens of pregnancy and childbirth. There may also be adverse consequences for children born to mothers who wanted to terminate their pregnancies and any existing children,^52^ though in retrospect some mothers may be glad they continued their pregnancies. A clinic-user who was dissuaded from having an abortion may not feel that she has been adversely affected because she believes she has changed her decision about the abortion. However, it is difficult to be certain that she genuinely and freely changed her decision after an encounter with demonstrators. If the decision was not genuine and freely made, this may itself be an adverse consequence.^53^

The adverse consequences for a clinic-user where an abortion is delayed are also well known. Indeed, she will be exposed to a greater risk of physical harm.^54^ This is because mortality and complications from abortion *increase* as pregnancy progresses.^55^ Additionally, she may have to undergo a more major surgical (as opposed to medical) abortion if the delay pushes her past a certain point in the pregnancy.^56^ Finally, clinic-users who do not want to be pregnant will likely find the delay to their abortions stressful.^57^

There are also adverse consequences for a clinic-user who encounters demonstrations but still goes ahead with the abortion that day. In *Dulgheriu*, the Court of Appeal referred to the ‘significant emotional and psychological damage’ endured by clinic-users who had been exposed to the demonstrations.^58^ Indeed, there is evidence that the demonstrations evoke an emotional reaction in clinic-users. Researchers who analysed comment forms completed by clinic-users between August 2011 and April 2015 at various British Pregnancy Advisory Service (BPAS) clinics in England and Wales found that many respondents used descriptors such as ‘upset, intimidated, uncomfortable, distressed and stressed’ to describe their encounters with demonstrators.^59^ Some respondents also reported feeling angry.^60^ Crucially, the researchers did not include clinics where more ‘extreme’ activities (such as the display of graphic images) occurred.^61^ Rather, they focussed on clinics where prayer-vigils and pavement counselling were common.^62^ This suggests that it is difficult to categorise particular activities as more or less bad. Indeed, the researchers propose that ‘the harassment that [clinic-users] feel … stems from the *presence* of activists at clinic sites, rather than from their precise conduct’.^63^ Research from the USA suggests that clinic escorts may help to reduce the negative emotional response endured by clinic-users, but this is not a common practice in England and Wales.^64^

There is conflicting evidence, however, about the ‘longevity’ of clinic-users’ negative emotional responses. Some research from the USA has found that demonstrations generally ‘do not affect [clinic-users’] subsequent feelings about their abortions’ and that clinic-users do not usually endure ‘long term negative psychological effects’.^65^ In contrast to this is Turner J’s unappealed finding (when *Dulgheriu* was in the High Court) that the activities that took place outside the abortion clinic were ‘fully capable of having a detrimental effect on the quality of life’ of clinic-users visiting clinics, and—crucially—that this was likely to be of a ‘persistent or continuing nature’.^66^

There is only very limited research on whether clinic-users are likely to suffer from recognised mental health conditions as a result of their encounters with demonstrators.^67^ In a small study conducted in the USA, twenty-five clinic-users completed an IES-R survey to measure post-traumatic stress events 7 days after their visit to an abortion clinic affected by demonstrations in Kentucky.^68^ The majority had normal IES-R scores, but 48% of participants had scores that either indicated concern for Post-traumatic Stress Disorder or probable Post-traumatic Stress Disorder.^69^ Other research has shown that abortion itself does not increase a clinic-user’s risk of mental health problems.^70^

The next step is to determine whether these adverse consequences constitute harm for the purposes of the Harm Principle. Unhelpfully, there is no single agreed definition of harm.^71^ Nevertheless, many commentators have interpreted Mill’s work as making a distinction between harm and mere offence.^72^ This is to say that an ‘individual’s emotional distress or expression of dislike’ is not harm.^73^ This might be problematic for the justification of buffer zone legislation because the *only* adverse consequence of the demonstrations for some clinic-users is a negative emotional reaction. It may be significant though that these clinic-users *might have* suffered some physical or mental injury as a result of the demonstrations; they just happened not to suffer any physical or mental injury.

For other philosophers, however, the Harm Principle is not the *only* valid principle relevant to when States can legitimately criminalise conduct. Feinberg, for example, defends a ‘rigorously qualified’ version of the Offence Principle.^74^ As the name suggests, this principle maintains that criminalising conduct can be justified to prevent offence (which Feinberg defines as ‘the whole miscellany of universally disliked mental states’).^75^ However, criminalisation will only be justified, according to Feinberg, where the seriousness of the offence outweighs the reasonableness of the offending conduct.^76^ This balancing test will now be applied in the context of ‘general clinic protest’ to establish whether fixed buffer zone legislation could be justified using Feinberg’s Offence Principle.

To do this, the seriousness of the mental distress endured by clinic-users seeking an abortion who are exposed to the demonstrations must be established. For the purpose of this analysis, the clinic-users must be ‘normal’ clinic-users seeking an abortion rather than clinic-users with ‘abnormal susceptibilities’ to offence.^77^ Helpfully, Feinberg provides a number of ‘standards’ which can be applied to determine the seriousness of the offence.^78^ The first of these is the ‘magnitude of the offence, which is a function of its intensity, duration, and extent’.^79^ It has already been noted that demonstrations induce intense feelings (upset, intimidation, discomfort, distress, stress, and anger) as opposed to ‘weak annoyance’.^80^ Further, it seems reasonable to suppose that the demonstrations ‘would offend nearly any [clinic-user seeking an abortion]’.^81^ This is not to say that all clinic-users seeking an abortion would feel the same way about the demonstrations (some might be upset whilst others might be angry) but it is difficult to imagine that many clinic-users would be unaffected. Whether the mental distress caused by the demonstrations is enduring is more uncertain given the conflicting evidence considered above, but the Court of Appeal found that it was in *Dulgheriu*.^82^

Feinberg’s second ‘standard’ is that of ‘reasonable avoidability’.^83^ It would be impossible for a clinic-user seeking an abortion to avoid the offence caused by the demonstrations because they occur outside the entrance to the abortion clinic they are trying to enter.^84^ A clinic-user seeking an abortion might be able to travel to another abortion clinic which is unaffected by demonstrations on that particular day, but it is difficult to know where demonstrations are occurring and, as noted above, they are becoming increasingly common. Even if there were another clinic unaffected by demonstrations, travelling to an alternative clinic would be a significant ‘inconvenience’ for clinic-users seeking an abortion.^85^

Feinberg’s third ‘standard’ is the ‘Volenti Maxim’, which refers to ‘offended states that were voluntarily incurred’.^86^ It might be argued that a clinic-user ‘voluntarily assumes’ the risk of offence by choosing to attend an abortion clinic, knowing that demonstrations may occur there.^87^ Telemedical early abortion, which allows pregnant women and other pregnant persons to take abortion medication at home up to 10 weeks of pregnancy, is due to continue beyond the Covid-19 pandemic as a result of a forthcoming amendment to the Abortion Act 1967.^88^ Nevertheless, some individuals will still have no choice but to visit a clinic in order to (lawfully) terminate their pregnancy. They may have passed the 10-week limit, or they may not meet medical safety criteria for remote consultation. Therefore, the maxim is not invoked here. Having applied Feinberg’s ‘standards’ to the offence endured by normal clinic-users seeking an abortion, it is apparent that the offence is very serious.

Next, the reasonableness of the demonstrations outside abortion clinics must be established. Again, Feinberg provides a number of ‘standards’ which can be applied to determine the reasonableness of the offending conduct.^89^ The first of these is ‘the importance of the conduct to the actor’.^90^ Presumably, demonstrating outside abortion clinics is of great ‘personal importance’ to demonstrators, for whom opposing abortion is a matter of moral and/or religious conviction.^91^ Feinberg’s second ‘standard’ is the ‘social utility’ of the conduct.^92^ Whether opposing abortion benefits the public is highly contentious, but the public value of freedom of expression is clear.^93^ Feinberg would regard freedom of expression to be especially valuable in this context because the demonstrations express opinions on ‘scientific, theological, philosophical, political, and moral questions’ relating to abortion.^94^

Feinberg’s third ‘standard’ is ‘the availability of other opportunities’.^95^ The demonstrators could demonstrate elsewhere to avoid causing offence to clinic-users accessing the abortion clinic.^96^ However, moving demonstrators away from abortion clinics may cause them ‘loss or unreasonable inconvenience’ because this location allows them to reach clinic-users accessing abortion services.^97^ Feinberg’s fourth ‘standard’ concerns a ‘spiteful or malicious … impelling motive’.^98^ Presumably, ‘malice and spite’ does not motivate the majority of demonstrators. This is to say that they are not offending clinic-users seeking an abortion ‘for the sake of it’.^99^ Rather, they are motivated by their moral and/or religious opposition to abortion.^100^ Even if demonstrators are aware of the offence they are causing,^101^ it seems reasonable to assume that most act ‘despite this’ not ‘because of it’.^102^

Feinberg’s final ‘standard’ is the ‘nature of the locality’.^103^ According to Feinberg, demonstrations will be more reasonable where they regularly occur and this is well known than where they do not.^104^ This standard therefore applies differently to each abortion clinic, depending on whether it is a ‘hotspot’ for demonstrations. This exposes a weakness inherent in the standard because conduct which occurs more frequently in a particular area is not necessarily *accepted* there. Rather, it may be seen as a problem. Whether or not conduct is accepted in the locality may be a better measure of reasonableness, therefore. Nevertheless, the conduct of demonstrators may be very reasonable on Feinberg’s account.

The final stage of the analysis is to balance the seriousness of the mental distress endured by clinic-users against the reasonableness of the demonstrations. This is a ‘hard case’ because the offence is very serious and the offending conduct is very reasonable,^105^ so either the seriousness of the offence or the reasonableness of the conduct could prevail. As Feinberg notes, ‘there [is] no automatic mathematical way of coming to a clearly correct decision’.^106^ Ultimately, the decision is a matter of judgement.^107^ But the conclusion reached here (that it could go either way) is nevertheless significant because it means that a legislative decision to criminalise protest activities outside abortion clinics is not obviously unjustified on Feinberg’s account.

Existing legislation will now be examined to determine whether it could be used to restrict demonstrations outside abortion clinics, starting with the Public Order Act (POA) 1986. Section 14(1) of the POA allows the senior police officer to impose conditions on a public assembly (including where it may be held) if he ‘reasonably believes that (a) it may result in serious public disorder, serious damage to property or serious disruption to the life of the community, or (b) the purpose of the persons organising it is the intimidation of others with a view to compelling them not to do an act they have a right to do’. Given the typical protest activities undertaken outside clinics in England and Wales, it seems unlikely that the demonstrations will lead to serious public disorder or serious damage to property. This can be contrasted with the USA, where there are numerous examples of abortion clinics being damaged.^108^ The consultation cited in *Dulgheriu* suggests that local residents and passers-by found the demonstrations stressful and upsetting,^109^ but it is not clear that this would be regarded as serious disruption to the life of the community. Indeed, section 14(1)(a) of the POA is typically relied on where transport is delayed.^110^ The delay to some clinic-users’ abortions may be closer to this, but only a small section of the community is affected in this way. Therefore, section 14(1)(a) of the POA is unlikely to be helpful in this context. Section 14(1)(b) of the POA is also unhelpful. Demonstrators want to prevent clinic-users going through with an abortion, but they would surely deny that they intend to achieve this by ‘intimidating’ clinic-users.^111^

Another option is section 5(1) of the POA which provides that ‘a person is guilty of an offence if he (a) uses threatening or abusive words or behaviour, or disorderly behaviour, or (b) displays any writing, sign or other visible representation which is threatening or abusive, within the hearing or sight of a person likely to be caused harassment, alarm or distress …’. Section 5(1) of the POA may be relevant to particular activities undertaken by the demonstrators, rather than the demonstration as a whole. Section 5(1)(a) may catch shouting at and/or approaching a clinic-user, for example, whilst section 5(1)(b) may catch displaying graphic images. Whether silent prayer would be caught by either of these subsections is more contentious, however. Even where an activity is caught by section 5(1) of the POA, the demonstrator undertaking it is unlikely to be guilty of an offence because they must have intended their actions to be threatening or abusive or be aware of this.^112^ This mental element was not satisfied in *DPP v Clarke*, where the defendants had displayed photographs of ‘aborted foetuses’ outside an abortion clinic.^113^ Today, the defendants in *Clarke* would also have been able to invoke free speech protection.^114^

A wider survey of the law unearths other offences that could be used to respond to particular activities undertaken by demonstrators outside abortion clinics. However, it is not clear that the criteria for these offences would be satisfied either. The display of graphic images outside abortion clinics, for example, might be an offence under section 1(1)(b) of the Indecent Displays (Control) Act 1981 which provides that ‘if any indecent matter is publicly displayed the person making the display and any person causing or permitting the display to be made shall be guilty of an offence’. However, it is not clear whether the graphic images would constitute *indecent* matter. The statute was enacted to deal with public displays of pornographic material, which is of a very different nature to images of foetuses.^115^ The term ‘indecent’ is not defined by the legislation, so it would likely be given its ordinary meaning.^116^ In *Connolly v DPP*, Dyson LJ found that ‘close-up colour photographs of dead 21-week-old foetuses’ were indecent for the purposes of section 1(1)(b) of the Malicious Communications Act,^117^ but with some ‘hesitation’.^118^

The common law offence of outraging public decency may provide an alternative route to restrict the display of graphic images outside abortion clinics.^119^ Indeed, a conviction for outraging public decency was upheld by the Court of Appeal where earrings ‘made from a freeze-dried human foetus of three to four months’ had been put on public display in an art gallery.^120^ Nevertheless, the display of an actual foetus may outrage the public to a greater extent than mere images of a foetus, especially as the appellant had tampered with the foetus by attaching a ring fitting to it.^121^ It is therefore unclear whether any public outrage regarding an image of a foetus would be sufficient.

The House of Lords found that the British Broadcasting Corporation had acted lawfully in declaring that a party election broadcast by the Pro-Life Alliance, which included graphic images of aborted foetuses, would be offensive to public feeling.^122^ This is a distinct but similar test to outraging public decency, so the decision provides further indication as to how the courts might approach the display of graphic images. Lord Hoffman observed that individuals who had previously had an abortion would be particularly likely to find the images offensive.^123^ Clinic-users who have just had or are about to have an abortion would be in a similar position. Lord Nicholls spoke to the fact that the use of graphic images was excessive, which suggests that ‘strictly limited’ use by demonstrators may be acceptable.^124^

Distributing leaflets containing false information outside abortion clinics might be an offence under section 1(1)(a) of the Malicious Communications Act 1988, providing distribution can be regarded as ‘sending’/‘delivering’ the leaflets.^125^ An example of the false information included in these leaflets is that there is a connection between abortion and breast cancer.^126^ Medical evidence undermines this claim.^127^ However, the mental elements of the offence may not be satisfied by demonstrators. First, a demonstrator may not know or believe that the information in the leaflets is false.^128^ Second, the intentions of a demonstrator may be to help clinic-users, rather than to cause them anxiety or distress.^129^

Even if the elements of the offences under section 5(1) of the POA, or section 1(1)(b) of the Indecent Displays (Control) Act, or section 1(1)(a) of the Malicious Communications Act could be satisfied, these offences would only deal with *some* of the varied activities undertaken by demonstrators outside abortion clinics. Indeed, this legislation could not respond to the presence of demonstrators or the demonstrations as a whole.

Perhaps the offence of public nuisance, which is due to be put on a statutory footing, would be more effective in this regard. Demonstrators’ actions, broadly construed, put a section of the public (clinic-users and clinic staff) at risk of suffering ‘serious distress, serious annoyance, serious inconvenience or serious loss of amenity’.^130^ Even if a demonstrator did not intend these consequences, he may have been reckless if he was aware that they might occur.^131^ The bigger obstacle will be the reasonable excuse defence,^132^ because the Law Commission has suggested that this would apply where an individual was exercising his rights under Articles 10 and 11 ECHR.^133^

Most of the demonstrations occur on the street outside abortion clinics. As such, demonstrators are not trespassing on the clinic’s land and they cannot be guilty of aggravated trespass.^134^ Demonstrators may be criminally liable for wilfully obstructing the highway though, providing they do not have a lawful excuse.^135^ A demonstrator will have a lawful excuse where the interference with his ECHR rights by the public authority was not proportionate.^136^ The UKSC has recently stressed that ‘there should be a certain degree of tolerance to disruption … caused by the exercise of’ rights under Articles 10 and 11 ECHR, and that there must be an assessment of the facts to establish whether the interference was proportionate.^137^ Providing that the demonstrations outside clinics (i) are peaceful, (ii) do not involve the commission of other offences, (iii) are targeted at clinic-users and clinic staff only, (iv) do not completely obstruct the highway, and (v) are of limited duration, the interference will not be proportionate and the demonstrators will have a defence.^138^

Another relevant piece of legislation is the Protection from Harassment Act (PHA) 1997. Harassment is defined by the statute as causing a person alarm or distress.^139^ It would have been more difficult to rely on the PHA in relation to demonstrations outside abortion clinics before 2005 because ‘a course of conduct which amounts to harassment of another’ for the purposes of section 1(1) of the PHA required ‘conduct on at least two occasions in relation to that person’.^140^ A clinic-user’s visit to the clinic appears to be just one occasion (unless her entrance and exit can be treated separately) and it seems unlikely that the same two individuals would encounter each other again if the clinic-user returned to the clinic on another occasion. Clinic staff might be more likely to experience at least two incidents by the same demonstrator or group of demonstrators, however, because they visit the clinic on a regular basis.

Since the PHA was amended in 2005,^141^ it may (by chance) be more helpful in the context under consideration here. Indeed, ‘a course of conduct’ for the purposes of section 1(1A) requires conduct on just one occasion.^142^ This provision also requires the harassment of ‘two or more persons’,^143^ which means it can respond to the fact that a demonstrator may target multiple clinic-users entering a clinic. Further, the provision requires an intention to ‘persuade any person … not to do something that he is entitled … to do’.^144^ Here, this is persuading clinic-users not to go through with an abortion.

One possible obstacle to the utility of the provision in this context, however, is the requirement that the demonstrator ‘knows or ought to know’ that his/her conduct ‘involves harassment of those persons’.^145^ Indeed, a demonstrator may believe that he/she is merely offering clinic-users help, support, and alternatives to abortion without alarming or distressing them.^146^ The possibility that a demonstrator ‘ought to know’ may be helpful though, at least in relation to *some* activities. Many pro-life advocates agree, for example, that graphic images of foetuses are distressing.^147^

If this obstacle can be overcome such that the requirements of section 1(1A) of the PHA are satisfied, and/or section 1(1) of the PHA is satisfied, the demonstrator will be guilty of a criminal offence.^148^ However, this means that the provision deals with individual demonstrators only. In this way, the PHA seems to be an inefficient response to the demonstrations, which may involve a large number of demonstrators. Injunctive relief, which is available under the PHA, is also problematic in this regard.^149^ Further, applying for and renewing injunctions places a significant burden on clinic staff and/or clinic-users. There may be little point in clinic-users applying for an injunction in any event as they are unlikely to be targeted again unless they make another visit to the clinic. Clinic-users may also be reluctant to report incidents to the police, which is a problem for all the offences considered in this article.^150^

The final piece of legislation to be considered is the Antisocial Behaviour, Crime and Policing Act 2014, ^151^ which allows a local authority to make a PSPO in response to a specific instance of anti-social behaviour for an initial maximum period of 3 years.^152^ PSPOs have been used successfully to restrict demonstrations outside some abortion clinics so there is no need to consider in any detail whether the legislation could be applied in this context.^153^ However, it is worth noting that utilising PSPOs here seems to have required a generous interpretation of the legislation, in two respects, by the Court of Appeal in *Dulgheriu*. First, local authorities appear to have been given ‘a broad discretion’ to determine that the activities in question have had, or are likely to have, a ‘detrimental effect’ (‘on the quality of life of those in the locality’).^154^ As Bhogal and O’Leary have observed, ‘[t]here is no requirement … to consider only “objective” or “reasonable” detriment’.^155^ This means that local authorities can take into account the particular vulnerability of clinic-users visiting abortion clinics.^156^ Second, it has been found that ‘those in the locality’ includes clinic-users who visit the clinic just once or twice.^157^ A less strained interpretation of this phrase might be limited to ‘residents’ and ‘those who regularly visit or work in the locality’ (the local community), as the applicants unsuccessfully argued in *Dulgheriu*.^158^

Despite having been used successfully to restrict demonstrations outside some abortion clinics, PSPOs do not provide a satisfactory solution to the problem of ‘general clinic protest’. Indeed, PSPOs have been described by BPAS as a ‘helpful stopgap’ but ‘not a permanent solution’.^159^ PSPOs are not a satisfactory solution because the protection they afford has been described as being subject to a ‘postcode lottery’.^160^ This is to say that PSPOs may be effective at restricting demonstrations once in place, but only a small number of abortion clinics in England and Wales actually have one in place.^161^ Indeed, this number is significantly smaller than the number of clinics affected by ‘general clinic protest’.^162^

BPAS has suggested that one reason why so few clinics have PSPOs in place is that ‘irregular protests’ can be ‘difficult to fit under persistent anti-social behaviour laws’.^163^ It is true that a local authority cannot make a PSPO unless ‘the effect, or likely effect, of the activities is, or is likely to be, of a persistent or continuing nature’.^164^ However, this condition looks to the *effects* of the demonstrations rather than, as BPAS suggests, the demonstrations themselves. Moreover, the Court of Appeal in *Dulgerhiu* were willing to accept that the effects on the clinic-users satisfied this condition.^165^

The real reason why so few clinics have PSPOs in place seems to be that local authorities lack the time and resources to undertake the ‘onerous’ process of securing a PSPO.^166^ The problem of inadequate time and resources is exacerbated by the temporary nature of PSPOs, which means that they have to be renewed.^167^ This problem would be eliminated by national legislation which implemented fixed buffer zones outside all clinics in England and Wales automatically. Therefore, introducing national buffer zone legislation would be a proportionate response.

The inadequacy of existing legislation is perhaps illustrated most clearly by the fact that demonstrations are occurring and even increasing. In any event, the issue of demonstrations outside abortion clinics is important in its own right, which justifies a specific piece of law. Indeed, the reach of fixed buffer zone legislation is limited (i) geographically, to the vicinity of abortion clinics and (ii) to protest activities related to abortion. This should prevent the police using the legislation to suppress any other demonstrations. While there may be concern that such legislation would set a worrying precedent, demonstrations outside abortion clinics are unique due to the particular vulnerability of clinic-users (who find themselves in a difficult position, whether that is an unwanted pregnancy or a much-wanted pregnancy that is affected by medical issues) and the enduring nature of the demonstrations.

## III. THE RELATIONSHIP BETWEEN THE AIMS AND THE MEANS: THE NATURE OF THE PROHIBITION

The proportionality assessment will now move on to consider the nature of the prohibition (as defined by legislation). No fixed buffer zone legislation currently exists in England and Wales, but the Bill and the amendment offer some insight into what future legislation may look like. Three key aspects will be considered: (i) the size of the buffer zone, (ii) the activities restricted within the buffer zone, and (iii) the penalty for violating the prohibition.

First, the buffer zones are ‘large’.^168^ The buffer zones in both the Bill and the amendment would have extended 150m from the clinic,^169^ which (as Landrigan notes) is equivalent to the length of ‘three Olympic swimming pools’.^170^ Second, the restriction on activities is comprehensive. Indeed, both the Bill and the amendment would have restricted all conceivable forms of demonstration—even ‘persistent, continuous or repeated occupation’ within the buffer zone.^171^ Third, the penalty for violating the prohibition is substantial. Both the Bill and the amendment specified 6 months (maximum) imprisonment and/or an unlimited fine for a first offence.^172^ Therefore, the extent of the interference with the ECHR rights of demonstrators would be considerable if fixed buffer zone legislation defined buffer zones in the same way as the Bill and the amendment. Given this conclusion, the question is whether there are any less restrictive measures that would be equally effective.

The Australian High Court rejected any suggestion that a buffer zone smaller than 150m would be equally effective:

… one cannot say that a smaller safe access zone would be as effective in restricting the ability of those who wish to have their say about abortions in the presence of a captive audience of [clinic-users] and those involved in advising and assisting them, while at the same time imposing a lesser practical burden on the implied freedom.^173^

The Australian High Court does not explain this conclusion and it seems to be at odds with the fact that buffer zones in Canada are typically only 50m.^174^ However, the legislation in some Canadian provinces recognises that the buffer zones may need to be extended by regulation.^175^ This is also recognised by legislation in the Australian Capital Territory (the only region in Australia where buffer zones are not automatically 150m).^176^ Similarly, the buffer zones in the Northern Irish Bill are 100m and can be extended up to 150m.^177^ This suggests that the most appropriate size for a buffer zone depends on the location and circumstances of a particular abortion clinic. Landrigan makes a similar argument and provides an example of an abortion clinic with a ‘single 200m walkway … with no alternative pedestrian access’ where a buffer zone of 150m would, in his opinion, be ‘minimally necessary’.^178^

In all, 150m may have been chosen for the buffer zones as defined by the Bill and the amendment to ensure that the buffer zones would be sufficient for as many abortion clinics as possible, given that the Bill and the amendment would have applied automatically to *all* abortion clinics in England and Wales. However, this may mean that the law is doing more than is strictly necessary in relation to some abortion clinics where a smaller zone might be sufficient. Indeed, the ‘buffer zone’ at issue in *Dulgheriu* was only 100m.^179^ Perhaps, then, a more flexible tailored approach to the size of the buffer zone in future legislation is needed to ensure that the interference is proportionate to the aim. This could be achieved by the legislation setting out a large buffer zone (150m) as the default, accompanied by a provision that allows demonstrators to request a reduction in size for a particular clinic.

At the Australian High Court, Mrs Clubb argued that there were less burdensome alternatives with regards to the behaviour that was prohibited by the Victorian fixed buffer zone legislation.^180^ Indeed, she argued that mere ‘communication in relation to abortions’ (as opposed to ‘besetting, harassing, intimidating, interfering with, threatening, hindering, obstructing or impeding’ a person accessing/leaving a clinic) should not be prohibited.^181^ ‘Communication in relation to abortions’ approximately corresponds to ‘advising or persuading or otherwise expressing an opinion’ on abortion and ‘informing about abortion services by any means’ in the Bill and the amendment.^182^ However, the Australian High Court rejected this argument for two reasons. First, communication in relation to abortions was not exceptional behaviour. Indeed, the Australian High Court observed that this behaviour ‘may well be apt to shame or frighten [a clinic-user] into eschewing the services of a clinic’.^183^ Second, a similar approach to the one suggested by Mrs Clubb had been tried before without success. The Australian High Court noted that ‘… the laws in Victoria prior to the enactment of [the legislation] did not adequately protect [clinic-users] seeking to access reproductive health clinics …’.^184^ Similarly, existing legislation in England and Wales is not sufficient (as shown above).

The appellants in *R v Spratt* made a similar, but more specific, argument that ‘at a minimum prayer-vigils which do not block entry to the clinic and non-violent sidewalk counselling should fall outside the prohibitions’.^185^ The Supreme Court of British Columbia rejected this and stressed that it would be impossible to exempt non-violent sidewalk counselling in practice:

To try to characterize each individual approach to every [clinic-user] entering the clinic is too difficult a calculus when the intent of the legislation is to give unimpeded access to the clinic. Therefore, a clear rule against *any* interference is the best way to achieve the ends of the legislation.^186^

Further, Lowe and Hayes concluded that ‘only the complete removal of anti-abortion activists from outside clinics will suffice in removing the source of the distress’ experienced by clinic-users.^187^

The Supreme Court of British Columbia did not respond directly to the appellants argument about prayer-vigils, but these should not be exempted either given the statement of the Australian High Court that ‘[s]ilent but reproachful observance of persons accessing a clinic for the purpose of terminating a pregnancy may be as effective, as a means of deterring them from doing so, as more boisterous demonstrations’.^188^

Therefore, none of the behaviour that is prohibited by the Bill and the amendment should be permitted if the legislation is to be effective. It is worth noting, however, that the behaviour is only prohibited *within* the buffer zone. All the activities prohibited by the Bill and the amendment can be performed elsewhere, so they are not totally banned. This fact is also relevant to the proportionality analysis with regard to fixed buffer zone legislation.^189^

The size of the penalty for violating the prohibition in fixed buffer zone legislation has not been examined in the international jurisprudence. However, Walsh (writing about Tasmanian and Victorian law) suggests that a less ‘substantial’ penalty might be an ‘… alternative means of achieving the same purpose’ but with a ‘less restrictive effect on freedom’.^190^ The maximum prison term provided for a first offence by the Bill and the amendment (6 months) is half the length of those provided by Tasmanian and Victorian law (12 months),^191^ so Walsh might regard this as more proportionate. However, the Northern Irish Bill does not provide for imprisonment at all.

An unlimited fine is a much more substantial penalty than the fines provided for by Tasmanian and Victorian law, which are capped at 12,975 AUD and 21,808.30 AUD respectively, and the Northern Irish Bill.^192^ Of course, the mere fact that a fine *could* be any amount does mean that large fines will be imposed. The fine must reflect the seriousness of the offence (so could respond to more/less ‘extreme’ activities) and take into consideration the offender’s financial circumstances.^193^ Moreover, an unlimited fine for violating the prohibition in fixed buffer zone legislation is in line with the penalty for harassment without violence, which is a similar offence.^194^ As such, there is no need to move the offence down to a lower level on the standard scale for the fine to be proportionate.

Prison terms and fines are both criminal sanctions, but perhaps providing clinic-users with a civil remedy (therefore avoiding the force and stigma of the criminal law) would be sufficient. Fixed buffer zone legislation could empower clinic-users to bring action against demonstrators in the civil courts, which can grant injunctions (to prevent actual or anticipated prohibited behaviour) and damages (to compensate for any loss suffered as a result of encountering the prohibited behaviour). If the statute only provided a civil remedy, this may circumvent the debate above about whether clinic-users suffer harm. This is the apparent standard for criminalisation, although much of the legislation considered in this article is not limited to preventing harm in the way Javid envisaged. The debate may still be relevant, however, given that damages are not recoverable in Tort for mental distress.^195^ Nevertheless, the PHA expressly provides that damages may be awarded for the ‘anxiety’ caused by the harassment.^196^ Even so, placing the onus on an individual clinic-user to sue demonstrators would be a significant burden to impose given the time and money that would be required during a period when she is already dealing with a difficult situation. Further, merely requiring demonstrators to compensate for loss rather than to accept punishment may prevent the law operating as a deterrent to other demonstrators. This is hugely problematic for legislation which is intended to stop particular behaviour from occurring. Therefore, criminal sanctions should be provided for in fixed buffer zone legislation.

Now that less restrictive measures have been considered, it is important to note how the ECtHR might incorporate this into its analysis were it to examine fixed buffer zone legislation.^197^ This is because ‘[t]he affirmation of the less restrictive means principle as a general guideline for restrictions of Convention rights is a recent development in the Court’s case law’ and its application has been chaotic thus far.^198^ Three pertinent points must be made. First, the ECtHR may not take the less restrictive means principle into account because references to it ‘are not systematic in any line of case law to date’.^199^ However, the Court of Appeal did cite the less restrictive means principle in *Dulgheriu*.^200^ Second, the less restrictive means principle would likely be ‘one factor among several’ if the ECtHR were to apply it (explicitly or implicitly), as opposed to being ‘decisive in finding the disproportionality of a rights restricting measure’.^201^ Third, it is not clear which version of the less restrictive means principle would be applied as both ‘substantive’ (‘requiring domestic authorities to actually adopt less restrictive means’) and ‘procedural’ (‘requiring [domestic authorities] merely to ‘consider’ adopting such less restrictive measures’) have been applied ‘inconsistently’.^202^ Gerards has argued that the procedural version is preferable because it is difficult for courts to ‘establish equally effective alternatives’ and ‘determine what ‘least restrictive’ really means’.^203^ Rather than the ECtHR ‘[undertaking] extensive investigations of all possible hypothetical alternatives’ to determine whether the least restrictive approach had been adopted by fixed buffer zone legislation, then, the government would have to ‘demonstrate that the competent bodies have made a considerable effort to explore and evaluate various alternatives and to obtain sufficient information as to their hypothetical effects’.^204^

The government’s exploration and evaluation may have to be particularly serious in relation to fixed buffer zone legislation, due to the narrow margin of appreciation (discretion under the supervision of the ECtHR) they are likely to have in relation to Articles 10 and 11 ECHR.^205^ Indeed, the ECtHR has stressed that ‘there is little scope under [Article 10(2)] for restrictions on … debate of questions of public interest’,^206^ such as the abortion debate.^207^ The ECtHR has said that the same approach must be taken with regard to Article 11(2) ECHR.^208^ Nevertheless, there are reasons to doubt that demonstrators outside abortion clinics meaningfully contribute to the abortion debate. This article has already noted that the aim of demonstrators seems to be to affect the decisions of individual clinic-users to have an abortion, rather than to oppose law or policy in any effective way. Additionally, tactics such as making clinic-users feel guilty and relying on misinformation are not indicative of a robust debate. However, a similar argument was rejected by the Court of Appeal in *Dulgheriu* on the grounds that demonstrators expressed an opinion on a topic of public interest (abortion) in public.^209^ The court reached this conclusion despite the fact that the clinic-users ‘were the immediate target of those expressions of opinion’.^210^

Provided that fixed buffer zone legislation was to allow for some flexibility regarding the size of buffer zones and the buffer zones were otherwise the same as those defined by the Bill and the amendment, they would be proportionate.

## IV. THE RELATIONSHIP BETWEEN COMPETING INTERESTS

To complete the proportionality assessment, the relationship between the competing interests of demonstrators opposing abortion and clinic-users seeking an abortion will now be considered. This is necessary because the legitimate aim of the interference is likely to be the protection of the rights and freedoms of clinic-users.^211^ A detailed examination of the legitimate aim falls outside the parameters of this article.^212^ For the purposes of the proportionality assessment, it is sufficient to note that the Court of Appeal (in *Dulgerhiu*) found that clinic-users have a right under Article 8 ECHR ‘to access advice on abortion and medical procedures for abortion available under the laws of this country’ which is ‘clearly’ engaged where demonstrations occur outside abortion clinics.^213^ Therefore, the ECtHR must be satisfied that a fair balance has been struck between the rights of demonstrators and clinic-users.^214^ This balancing exercise will entail an examination of both the consequences of the demonstrations for clinic-users seeking an abortion and the consequences of buffer zones for demonstrators opposing abortion, so as to establish the ‘comparative importance of the rights being claimed’.^215^

The adverse consequences of demonstrations on clinic-users have already been discussed. In summary, a clinic-user may be dissuaded/deterred from having an abortion, her abortion may be delayed, and implementing her decision to have an abortion may be made more difficult. Consequently, a clinic-user’s physical and/or mental health may be negatively impacted. In contrast, the adverse consequences for demonstrators who are prevented from demonstrating within buffer zones are that they are unable to express their opinions in close proximity to abortion clinics. It seems unlikely that this would have a negative impact on demonstrators’ health, so this is the point of distinction.

In *Dulgheriu*, the Court of Appeal regarded physical/mental health as ‘important interests’ which clinic-users were ‘entitled’ to have protected.^216^ This was central to the court’s conclusion that a fair balance had been struck between the clinic-users’ and demonstrators’ rights.^217^ As Puppinck suggested when writing about balancing the competing rights of a pregnant woman and the individual who impregnated her, favouring the right of the pregnant woman (or other pregnant person) is more straightforward where her health is at risk.^218^ Indeed, it is intuitive. As such, it seems that fixed buffer zone legislation would strike a fair balance between the competing rights of clinic-users and demonstrators.

## V. CONCLUSION

This article has examined the proportionality of fixed buffer zone legislation. National legislation would be a proportionate response for the following reasons. First, an increasing number of clinics in England and Wales are now experiencing ‘general clinic protest’. Second, the non-violent nature of these demonstrations does not mean they do not warrant a response. Third, existing (civil and criminal) national legislation is unable to deal with the demonstrations satisfactorily. The nature of the prohibition would be proportionate, providing two conditions were satisfied: first, that the legislation allows for some flexibility regarding the size of the buffer zones; second, that the activities restricted and the penalties specified are the same as those defined by the Bill and the amendment. Fixed buffer zone legislation would strike a fair balance between the rights of clinic-users and demonstrators. This is because the health of clinic-users may be negatively affected by demonstrations, whereas buffer zones would not affect the health of demonstrators. In conclusion, national legislation which implements fixed buffer zones can be proportionate. Consequently, the ECHR is no impediment to the enactment of fixed buffer zone legislation in England and Wales.

## Footnotes

1 Department of Health and Social Care, ‘Abortion Statistics, England and Wales: 2020’ (Department of Health and Social Care 2021) para 4.6 <https://www.gov.uk/government/statistics/abortion-statistics-for-england-and-wales-2020/abortion-statistics-england-and-wales-2020> accessed 11 September 2021.

2 McGuiness has suggested that the standalone nature of abortion provision in England and Wales leaves clinics ‘vulnerable’ to demonstrations, see: Sheelagh McGuiness, ‘A Guerrilla Strategy for a Pro-Life England’ (2015) 7 Law Innov Technol 283, 293–94. However, some there have been demonstrations outside NHS hospitals too, see: BPAS, ‘Recorded Harassment Outside Clinics’ (*Back Off*, 2021) <https://back-off.org/recorded-harassment/> accessed 11 September 2021.

3 David Cohen and Krysten Connor, *Living in the Crosshairs: The Untold Stories of Anti-Abortion Terrorism* (OUP 2015) 6.

4 Police, Crime, Sentencing and Courts Bill Deb 24 June 2021, col 758.

5 HC Deb 24 June 2020, vol 677, cols 1314–1315; Maya Oppenheim, ‘Lawyers Threaten Council with Court Action to Provide ‘buffer zones’ Outside Abortion Clinics Amid Protests’ *The Independent* (17 May 2021) <https://www.independent.co.uk/news/uk/home-news/abortions-buffer-zones-harassment-women-b1847753.html> accessed 16 September 2021; *Dulgheriu and another v Ealing London Borough Council* [2019] EWCA Civ 1490, [2020] 1 WLR 609 [59]–[60].

6 Police, Crime, Sentencing and Courts Bill Deb 24 June 2021, cols 758–759.

7 Alesha Doan, *Opposition and Intimidation: Wars and Strategies of Political Harassment* (University of Michigan Press 2007) 133; Cohen and Connor (n 3) 6; Police, Crime, Sentencing and Courts Bill Deb 24 June 2021, col 758.

8 Doan (n 7) 133. McGuiness has suggested that ‘pro-life’ groups in the UK are making use of ‘guerrilla strategies’, including demonstrations outside abortion clinics, from the USA, see: McGuiness (n 2) 299.

9 Diana Foster and others, ‘Effect of Abortion Protests on Women’s Emotional Response to Abortion’ (2013) 87 Contraception 81, 86.

10 ibid. Therefore, the finding from some studies that interactions with demonstrators did not generally prevent clinic-users going ahead with the abortion should not be overstated. See for example: Christian Fiala, Johanna Özogul and Jennifer Kernreiter, ‘Experiences of Women Accessing an Abortion Clinic Confronted by Religiously Motivated Demonstrators: a Pilot Study’ (2020) 25 Eur J Contracept Reprod Health Care 228; Angel Foster, Mira Persaud and Kathryn LaRoche, ‘“I didn’t doubt my choice, but I felt bad”: A Qualitative Exploration of Canadian Abortion Patients’ Experiences with Protestors’ (2020) 102 Contraception 308; Erin Carrol and others, ‘Abortion Patient Experiences with Protestors While Accessing Care in Mississippi’ [2021] Sex Res Soc Policy 1.

11 Foster and others (n 9) 86.

12 Viv Rose, who managed BPAS’ Cardiff clinic told The Guardian newspaper that ‘Some people actually ring us and say ‘I’m not coming in because I’ve seen all the demonstrators outside. When do they go? And they rebook their abortion. That, to me, is dreadful because they’re not stopping people having abortions, they’re putting it off for the 40 days. So they’re actually making the women have abortions at later term than they normally would’. See: Simon Murphy ‘Anti-abortion Activists Harassing Women at UK Clinics during Lent’ *The Guardian* (8 April 2019) <https://www.theguardian.com/uk-news/2019/apr/08/anti-abortion-activists-harassing-women-at-uk-clinics-during-lent> accessed 22 September 2021. See also: HC Deb 13 September 2018 vol 646, col 36WS–38WS; *Dulgheriu* (n 5) [61].

13 RCOG and FSRH, ‘RCOG and FSRH Key Messages on Safe Abortion’ (*RCOG*) 3 <https://www.rcog.org.uk/en/global-network/centre-womens-global-health/our-work/making-abortion-safe/key-messages/healthcare/> accessed 22 September 2021.

14
*Re T* [1993] Fam 95, 113–114.

15 ibid.

16 In the Australian Capital Territory, however, the Health Minister has to declare a fixed buffer zone around an abortion clinic.

17 Abortion clinics may have to apply for a fixed buffer zone (British Columbia/Newfoundland and Labrador) or fixed buffer zones may only protect clinics set out in regulations (Nova Scotia/Alberta/Ontario).

18
*Clubb v Edwards*; *Preston v Avery* [2019] HCA 11; *R v Spratt* [2008] BCCA 340.

19 NIA, ‘Abortion Services (Safe Access Zones) Bill’ (*Non-Executive Bill Proposals*, 24 March 2022) <http://www.niassembly.gov.uk/assembly-business/legislation/2017-2022-mandate/non-executive-bill-proposals/abortion-services-safe-access-zones-bill/> accessed 26 March 2022.

20 Demonstrations (Abortion Clinics) HC Bill (2019–2021) [145]; Police, Crime, Sentencing and Courts Bill Deb 24 June 2021, col 757.

21 Convention for the Protection of Human Rights and Fundamental Freedoms (European Convention on Human Rights, as amended) arts 10(1) and 11(1).

22
*Van Den Dungen v The Netherlands* (1995) 80 DR 147, s 2.

23
*Dulgheriu* (n 5) [82] and [10].

24 ibid [82].

25 Convention for the Protection of Human Rights and Fundamental Freedoms (European Convention on Human Rights, as amended) art 9(1).

26
*Van Den Dungen* (n 22) complaints section; *Dulgheriu* (n 5) [8]; Pam Lowe and Sarah-Jane Page, ‘On the Wet Side of the Womb: The Construction of “mothers” in Anti-Abortion Activism in England and Wales’ (2019) 26 Eur JWomen’s Stud 165, 176.

27 Some demonstrators seem to ‘downplay’ their religious motivation and rely on ‘scientific facts’ instead, see: Lowe and Page (n 26) 175; Pam Lowe and Sarah-Jane Page, ‘Rights-based Claims Made by UK Anti-abortion Activists’ (2019) 21 Health Hum Rights J 133, 142.

28
*Van Den Dungen* (n 22) s 1.

29
*Arrowsmith v UK* (1978) 3 EHRR 218 (Commission Decision) paras 70–75.

30
*Van Schijndel v The Netherlands* App no 30936/96 (Commission Decision, 10 September 1997) s 1 and the facts section.

31 ibid s 1.

32
*Dulgheriu and another v Ealing London Borough Council* [2018] EWHC 1667, [2019] PTSR 706 [65].

33
*Dulgheriu* (n 5) [81].

34
*Eweida v United Kingdom* (2013) 57 EHRR 8, para 82; *Van Schijndel* (n 30) the facts section; *Van Den Dungen* (n 22) the facts section.

35 Pam Lowe and Graeme Hayes, ‘Anti-Abortion Clinic Activism, Civil Inattention and the Problem of Gendered Harassment’ (2019) 53 Sociology 330, 338. See further: Pam Lowe and Sarah-Jane Page, ‘Contested Embodiment: The Use of Prayer in Public Displays of Anti-abortion Activism’ in Sarah-Jane Page and Katy Pilcher (eds), *Embodying Religion, Gender and Sexuality* (Routledge 2021).

36
*Dulgheriu* (n 5) [80].

37
*Eweida* (n 34) para 82.

38
*Van Den Dungen* (n 22) s 1.

39
*Van Den Dungen* (n 22) the facts section. Religious iconography is widely used during demonstrations, see: Lowe and Page (n 26) 173.

40 Convention for the Protection of Human Rights and Fundamental Freedoms (European Convention on Human Rights, as amended) arts 9(2), 10(2) and 11(2).

41 HC Deb 13 September 2018 vol 646, col 37WS.

42 ibid.

43 Lowe and Hayes (n 35) 341; Lowe and Page (n 26) 166.

44 HC Deb 13 September 2018 vol 646, col 37WS.

45 ibid.

46 ibid.

47
*Clubb* (n 18) [89].

48 ibid. This was also noted by the Supreme Court of British Columbia, see: *Spratt* (n 18) [80].

49 HC Deb 13 September 2018 vol 646, col 37WS. Cf. Sarah Champion MP noted that, ‘much of the legislation has been thoroughly inadequate at addressing the problem [of “general clinic protest”]’ when she introduced the amendment to Parliament—see: Police, Crime, Sentencing and Courts Bill Deb 24 June 2021, col 759.

50 John Stuart Mill, *On Liberty* (first published 1859, Enhanced Media 2016) 12.

51 World Health Organization, *Mental Health Aspects of Women’s Reproductive Health* (WHO 2009) vii <http://apps.who.int/iris/bitstream/handle/10665/43846/9789241563567_eng.pdf;jsessionid=2C5234489B71F27DD37D9757227659DE?sequence=1> accessed 23 November 2021.

52 Diana Foster and Others, ‘Comparison of Health, Development, Maternal Bonding, and Poverty Among Children Born After Denial of Abortion vs Pregnancies Subsequent to an Abortion’ (2018) 172 JAMA Paediatr 1053; Diana Foster and others, ‘Effects of Carrying an Unwanted Pregnancy to Term on Women’s Existing Children’ (2019) 205 J Paediatr 183; BBC News, ‘Hythe Baby Death: Mother Wanted an Abortion, Court Hears’ *BBC News* (17 November 2021) <https://www.bbc.co.uk/news/uk-england-hampshire-59305514> accessed 21 November 2021.

53 Seana Valentine Shiffrin, ‘Harm and its Moral Significance’ (2012) 18 Legal Theory 357, 386; *Re T* (n 14) 113–14.

54
*Dulgheriu* (n 5) [61].

55 For medical evidence, see: Linda Bartlett and others, ‘Risk Factors for Legal Induced Abortion-Related Morality in the United States’ (2004) 103 Obstet Gynaecol 729, 736; David Grimes and Kenneth Schulz, ‘Morbidity and Mortality from Second Trimester Abortions’ (1985) 30 J Reprod Med 505. The risk of mortality and complications is still low, however, see: Patricia Lohr, Jonathan Lord and Sam Rowlands, ‘How Would Decriminalisation Affect Women’s Health?’ in Sally Sheldon and Kaye Wellings (eds), *Decriminalising Abortion in the UK: What Would It Mean?* (Policy Press 2020) 46.

56 At MSI Reproductive Choices UK, for example, medical abortion is only available up to 9 weeks + 6 days of pregnancy.

57 Julia Steinberg and others, ‘Psychological Factors and Pre-Abortion Psychological Health: The Significance of Stigma’ (2016) 150 Soc Sci Med 67, 75–76.

58
*Dulgheriu* (n 5) [61].

59 Lowe and Hayes (n 35) 341. See further: Pam Lowe and Graeme Hayes, ‘A Hard Enough Decision to Make: Anti-Abortion Activism outside Clinics in the Eyes of Clinic Users’ (Aston University 2015) <https://publications.aston.ac.uk/id/eprint/41856/1/A_Hard_Enough_Decision_to_Make.pdf> accessed 4 April 2022.

60 Lowe and Hayes (n 35) 341.

61 ibid 332–37.

62 ibid.

63 ibid 343 (emphasis added).

64 Catherine Cozzarelli and Brenda Major, ‘The Effects of Anti-Abortion Demonstrators and Pro-Choice Escorts on Women’s Psychological Responses to Abortion’ (1994) 13 J Clin Psychol 404, 423.

65 Foster and others (n 9) 86; Catherine Cozzarelli and others, ‘Women’s Experiences of and Reactions to Antiabortion Picketing’ (2010) 22 Basic Appl Soc Psychol 265, 274.

66
*Dulgheriu* (n 5) [54]–[55].

67 Richalle Sullivan, Tanya Franklin and Ashelee Bergin, ‘Anti-Abortion Picketing and Mental Health: Is There a Correlation Between Picketers and Post-traumatic Stress?’ (2020) 135 Obstet Gynaecol 93S.

68 ibid.

69 ibid.

70 See for example: Julia Steinberg and others, ‘Examining the Association of Antidepressant Prescriptions with First Abortion and First Childbirth’ (2018) 75 JAMA Psychiatry 828, 833.

71 A number of definitions have been proposed, see for example: Joel Feinberg, *The Moral Limits of the Criminal Law Volume 1: Harm to Others* (OUP 1984) 36 and 105–6; Jonathan Riley, *Mill on Liberty* (Routledge 1998) 99; John Gray, *Mill On Liberty: A Defence* (2nd edn, Routledge 1996) 57; David Brink, ‘Mill’s Liberal Principles and Freedom of Expression’ in Chin Liew Ten (ed), *Mill’s On Liberty: A Critical Guide* (CUP 2008) 42; Wendy Donner, ‘Autonomy, Tradition, and the Enforcement of Morality’ in Chin Liew Ten (ed), *Mill’s On Liberty: A Critical Guide* (CUP 2008) 161; David Dyzenhaus, ‘John Stuart Mill and the Harm of Pornography’ (1992) 102 Ethics 534, 546.

72 See for example: Jonathan Riley, ‘One Very Simple Principle’ (1991) 3 Utilitas 1, 6. Cf Piers Turner, ‘“Harm” and Mill’s Harm Principle’ (2014) 124 Ethics 299, 315.

73 Riley (n72) 6. Cf. Schwartz attempts to assimilate offence into harm, see: Louis Shwartz, ‘Morals Offences and the Penal Code’ (1963) 63 Columbia Law Rev 669, 671.

74 Joel Feinberg, *The Moral Limits of the Criminal Law Volume 2: Offense to Others* (OUP 1985) 46.

75 ibid 46 and 1.

76 ibid 25–49.

77 ibid 33.

78 ibid 34–35.

79 ibid 35.

80 ibid 27. See: Lowe and Hayes (n 35) 341.

81 Feinberg (n 74) 30–31.

82
*Dulgheriu* (n 5) [54]–[55]. Cf Foster and others (n 9) 86; Cozzarelli and others (n 65) 274.

83 Feinberg (n 74) 35.

84 ibid 32.

85 ibid.

86 ibid 35.

87 ibid 32–33.

88 Abortion Act 1967, s 1A.

89 Feinberg (n 74) 44.

90 ibid.

91 ibid 37; Lowe and Page (n 26) 176.

92 Feinberg (n 74) 44.

93 ibid 38.

94 ibid 44.

95 ibid.

96 ibid 40.

97 ibid; *Spratt* (n 18) [78].

98 Feinberg (n 74) 44.

99 ibid 41.

100 ibid; Lowe and Page (n 26) 176.

101 Lowe and Hayes (n 35) 338–40.

102 Feinberg (n 74) 41.

103 ibid 44.

104 ibid 42–44.

105 ibid 45.

106 ibid.

107 ibid.

108 The damage includes vandalism, arson, bombing and being driven into.

109
*Dulgheriu* (n 5) [47]–[51].

110 See for example: *R (on the application of Jones) v Commissioner of Police of the Metropolis* [2019] EWHC 2957, [2020] 1 WLR 519 [34].

111 Lowe and Hayes (n 35) 338–40.

112 Public Order Act 1986, s 6(4); Lowe and Hayes (n 35) 338–40.

113
*DPP v Clarke and Others* (1992) 94 Cr App R 359, 364 and 366. See also: Tania Branigan, ‘CPS Drops Case against Anti-Abortion Protestor’ *The Guardian* (10 July 2004) <https://www.theguardian.com/uk/2004/jul/10/ukcrime.taniabranigan> accessed 25 November 2021; Sara Boseley, ‘Anti-abortion Protestors Cleared of Public Order Offences’ *The Guardian* (17 September 2012) <https://www.theguardian.com/uk/2012/sep/17/anti-abortion-protesters-cleared> accessed 4 April 2022.

114 See for example: *Percy v DPP* [2001] EWHC Admin 1125, [2002] Crim LR 835.

115 HC Deb 30 January 1981, vol 997, cols 1166–1167.

116
*Brutus v Cozens* [1973] AC 854, 861; *Connolly v DPP* [2007] EWHC 237, [2008] 1 WLR 276 [10].

117 ‘A person who sends another person … any article or electronic communication which is, in whole or part, of an indecent or grossly offensive nature is guilty of an offence if his purpose … in sending it is that it should cause distress or anxiety to the recipient ….’ In *Connolly* (n 116), the appellant had sent pharmacists (who provided emergency contraception) images of aborted foetuses.

118
*Connolly* (n 116) [11].

119 ‘… if the offence of outraging public decency were to be proved, it was necessary to prove two elements. (i) The act was of such a lewd character as to outrage public decency; this element constituted the nature of the act which had to be proved before the offence could be established; (ii) it took place in a public place and must have been capable of being seen by two or more persons who were actually present, even if they had not actually seen it.’ See: *R v Hamilton* [2007] EWCA Crim 2062, [2008] QB 224 [21].

120
*R v Gibson* [1990] 2 QB 619, 630.

121 ibid 622.

122
*R v BBC ex parte Pro-Life Alliance* [2003] UKHL 23, [2004] 1 AC 185 [16] and [79].

123 ibid [80].

124 ibid [13].

125 Malicious Communications Act 1988, s 1(3).

126 Police, Crime, Sentencing and Courts Bill Deb 24 June 2021, col 758. For an example of a leaflet making this claim, see the evidence Sister Supporter provided Ealing Council as part of the PSPO application process: Sister Supporter, ‘Evidence—Leaflets’ (*Sister Supporter*) <https://www.sistersupporter.co.uk/evidence> accessed 18 October 2021.

127 See for example: Collaborative Group on Hormonal Factors in Breast Cancer, ‘Breast Cancer and Abortion: Collaborative Reanalysis of Data from 53 Epidemiological Studies, including 83,000 Women with Breast Cancer from 16 Countries’ (2004) 363 Lancet <https://doi.org/10.1016/S0140-6736(04)15835-2> accessed 18 October 2021.

128 Malicious Communications Act 1988, s 1(1)(a)(iii).

129 ibid s 1(1); Lowe and Hayes (n 35) 338–40.

130 Police, Crime, Sentencing and Courts HL Bill (2021–22) 95, cl78(1)(b) and cl78(2)(c).

131 ibid cl78(1)(c).

132 ibid cl78(3).

133 Law Commission, ‘Simplification of Criminal Law: Public Nuisance and Outraging Public Decency’ (Law Commission 2015) para 3.61 <https://www.lawcom.gov.uk/project/simplification-of-the-criminal-law-public-nuisance-and-outraging-public-decency/> accessed 30 March 2022.

134 Criminal Justice and Public Order Act 1994, s 68.

135 Highways Act 1980, s 137.

136
*DPP v Ziegler* [2021] UKSC 23, [2022] AC 408 [10]–[16].

137 ibid [68]–[70].

138 ibid [71]–[78].

139 Protection from Harassment Act 1997, s 7(2).

140 ibid s 7(3)(a).

141 Serious Organised Crime and Police Act 2005, s 125(2)(a).

142 Protection from Harassment Act 1997, s 7(3)(b).

143 ibid s 1(1A)(a).

144 ibid s 1(1A)(c).

145 ibid s 1(1A)(b).

146 ibid s 7(2); Lowe and Hayes (n 35) 338–40.

147
*Christian Hacking v London Borough of Waltham Forest* (Stratford Magistrates’ Court, 6 May 2020) [14]; *BBC* (n 122) [11].

148 Protection from Harassment Act 1997, s 2(1).

149 ibid s 3 and s 3A.

150 Olivia Petter, ‘Stalking, “lies” and Harassment: The Fight to Enforce Buffer Zones Outside Abortion Clinics’ *The Independent* (7 November 2019) <https://www.independent.co.uk/life-style/women/abortion-clinic-buffer-zones-uk-home-office-review-bpas-marie-stopes-london-a9188041.html> accessed 4 April 2022.

151 HC Deb 13 September 2018 vol 646, col 37WS.

152 Anti-Social Behaviour, Crime and Policing Act 2014, s 59(1) and s 60(1) (ASBCPA). Note that breaching a PSPO is a criminal offence—ASBCPA 2014, s 67(1).

153 HC Deb 13 September 2018 vol 646, col 37WS. See also: *Dulgheriu* (n 5).

154 Kuljit Bhogal and Tara O’Leary, ‘Public Spaces Protection Orders: What Have We Learned?’ (2019) 22 J Hous Law 7, 8; Ben Stanford, ‘Power to the People! Public Spaces Protection Orders and the Devolution of the Preventative State’ [2020] Public Law 719, 732.

155 Bhogal and O’Leary (n 154) 8.

156
*Dulgheriu* (n 5) [61].

157 Bhogal and O’Leary (n 154) 8; ibid [49].

158
*Dulgheriu* (n 5) [29] and [38].

159 BPAS, ‘Demonstrations (Abortion Clinics) Bill: Bill Briefing on Abortion Clinic Protests’ (2020) <https://www.bpas.org/media/3355/demonstrations-abortion-clinics-bill-briefing.pdf> accessed 5 October 2021.

160 ibid; Police, Crime, Sentencing and Courts Bill Deb 24 June 2021, col 759.

161 Police, Crime, Sentencing and Courts Bill Deb 24 June 2021, col 759.

162 ibid.

163 BPAS (n 159).

164 Antisocial Behaviour, Crime and Policing Act 2014, s 59(3)(a).

165
*Dulgheriu* (n 5) [44].

166 HC Deb 24 June 2020, vol 677, cols 1314–1315.

167 ibid; Antisocial Behaviour, Crime and Policing Act 2014, s 60.

168 Mitchell Landrigan, ‘Protests Outside Abortion Clinics: Constitutionally Protected Speech?’ (2016) 41 Altern Law J 8, 11. Cf The Australian High Court regarded the ‘burden on the implied freedom … in respect of … its geographical extent’ to be only ‘slight’—see: *Clubb* (n 18) [100].

169 Demonstrations (Abortion Clinics) Bill (n 20) cl 1; Police, Crime, Sentencing and Courts Bill Deb 24 June 2021, col 757.

170 Landrigan (n 168) 11.

171 Demonstrations (Abortion Clinics) Bill (n 20) cl 2(2); Police, Crime, Sentencing and Courts Bill Deb 24 June 2021, col 757.

172 Demonstrations (Abortion Clinics) Bill (n 20) cl 2(3)(a); Police, Crime, Sentencing and Courts Bill Deb 24 June 2021, col 757.

173
*Clubb* (n 18) [100].

174
*An Act Respecting Health and Social Services* CQLR 2021, cS-4.2 (as amended by *An Act to extend the powers of the Régie de l’assurance maladie du Québec, regulate commercial practices relating to prescription drugs and protect access to voluntary termination of pregnancy services* SQ 2016, c28, s 76 and s 79), s 16.1 [Quebec] (ARHSS 2021); *Safe Access to Abortion Services Act* SO 2017, c19, s 6(2) [Ontario] (SAASA 2017); *Protecting Choice For Women Accessing Healthcare Act* SA 2018, cP-26.83, s 6(2) [Alberta] (PCWAHA 2018); *An Act to Protect Access to Reproductive Health Care* SNS 2020, c5, s 7(2) [Nova Scotia] (APARHC 2020); *Access to Abortion Services Act* SNL 2016, cA-1.02, s 6(2) [Newfoundland and Labrador] (AASA 2016); *Access to Abortion Services Act* RSBC 1996, c1, s 5(2) [British Columbia] (AASA 1996).

175 SAASA 2017 s 6(2); PCWAHA 2018 s 6(4); APARHC 2020 s 16(1); AASA 2016 s 6(4); AASA 1996 s 5(4).

176
*Health Act* 1993 (ACT) (as amended by the *Health (Patient Privacy) Amendment Act* 2015 (ACT) s 5 and *Health (Improving Abortion Access) Amendment Act* 2018 (ACT)) s 86(3) (HA 1993). See also: *Termination of Pregnancy Act* 2018 (QLD) s 14(2) (TPA 2018); *Reproductive Health (Access to Terminations) Act* 2013 (TAS) s 9(1) (RH(AT)A 2013); *Reproductive Health Amendment (Safe Access to Reproductive Health Clinics) Act* 2018 (NSW) s 98A (RHA(SARHC)A 2018); *Public Health Act* 2016 (WA) (as amended by the *Public Health Amendment (Safe Access Zones) Act* 2021 (WA) s 4) s202O (PHA 2016); *Health Care Act* 2008 (SA) (as amended by the *Healthcare (Safe Access) Amendment Act* 2020 (SA) s 4) s 48B (HCA 2008); *Public Health and Wellbeing Act* 2008 (VIC) (as amended by the *Public Health and Wellbeing Amendment (Safe Access Zones) Act* 2015 (VIC) s 5) s 185B(1) (PHWA 2008); *Termination of Pregnancy Law Reform Act* 2017 (NT) s 4 (TPLRA 2017).

177 Abortion Services (Safe Access Zones) NIA Bill 35/17-322, cl4(2)-cl4(3).

178 Landrigan (n 168) 11.

179
*Dulgheriu* (n 5) [10].

180
*Clubb* (n 18) [87]–[88]. See: PHWA 2008 s 185B(1).

181
*Clubb* (n 18) [88]–[89].

182 Demonstrations (Abortion Clinics) Bill (n 20) cl2(2)(e) and cl2(2)(f); Police, Crime, Sentencing and Courts Bill Deb 24 June 2021, col 757. Cf Demonstrations (Abortion Clinics) Bill (n 20) cl2(2)(a)-(d).

183
*Clubb* (n 5) [88].

184 ibid [90].

185
*Spratt* (n 18) [77].

186 ibid [80].

187 Lowe and Hayes (n 35) 344.

188
*Clubb* (n 18) [89].

189 ibid [102].

190 Greg Walsh, ‘The Constitutionality of Communication Prohibitions Around Abortion Clinics’ [2018] West Australian Jurist 80, 101.

191 RH(AT)A 2013 s9(2); PHWA 2008 s185D.

192 ibid; Abortion Services (Safe Access Zones) NIA Bill 35/17-322, cl5(4).

193 Sentencing Act 2020, s 125.

194 Protection from Harassment Act 1997, s 2(2).

195
*Hinz v Berry* [1970] 2 QB 40, 42. See: Eric Descheemaeker, ‘Rationalising Recovery for Emotional Harm in Tort Law’ (2018) 134 LQR 602; Paula Giliker, ‘A “new” head of damages: damages for mental distress in the English law of torts’ (2000) 20 Legal Stud 19; Harvey Teff, *Causing Psychiatric and Emotional Harm: Reshaping the Boundaries of Legal Liability* (Hart Publishing 2009).

196 Protection from Harassment Act 1997, s 3(2).

197 Cf the approach of the US Supreme Court in McCullen v Coakley 134 S Ct 2518 (2014): the fixed buffer zone legislation in question was regarded as ‘content neutral’, so it did not need to be the least restrictive means of serving the government’s interests (2535).

198 Eva Brems and Laurens Lavrysen, ‘Don’t Use a Sledgehammer to Crack a Nut: Less Restrictive Means in the Case Law of the European Court of Human Rights’ (2015) 15 Hum Rights Law Rev 139, 166–68; Laurens Lavrysen, ‘On Sledgehammers and Nutcrackers: Recent Developments in the Court’s Less Restrictive Means Doctrine’ (*Strasbourg Observes*, 20 June 2018) <https://strasbourgobservers.com/2018/06/20/on-sledgehammers-and-nutcrackers-recent-developments-in-the-courts-less-restrictive-means-doctrine/> accessed 12 October 2021. For cases that explicitly affirm the less restrictive means principle, see: *Mouvement Raëlien Suisse v Switzerland* [GC] 2012-IV 373, para 75; *Nada v Switzerland* [GC] 2012-V 213, para 183; *Glor v Switzerland* 2009-III 33, para 94.

199 Brems and Lavrysen (n 198) 166.

200
*Dulgheriu* (n 5) [82].

201 Brems and Lavrysen (n 198) 166–67. The less restrictive means principle is one of four aspects of Lord Sumption’s test of proportionality, see: *Bank Mellat v Her Majesty’s Treasury (No 2)* UKSC 39, [2014] AC 700 [20].

202 ibid 167; Lavrysen (n 198).

203 Janneke Gerards, ‘How to Improve the Necessity Test of the European Court of Human Rights’ (2013) 11 Int J Constit Law 266, 486 and 489.

204 ibid 487.

205 ibid.

206
*Wingrove v UK* (1996) 24 EHRR I, para 58; *Animal Defenders International v UK* [GC] ECHR 2013-II 203, para 102.

207
*Annen v Germany* App no 3690/10 (ECtHR, 26 November 2015) para 64.

208
*Primov and Others v Russia* App no 17391/06 (ECtHR, 12 June 2014) paras 134–35.

209
*Dulgheriu* (n 5) [92].

210 ibid.

211
*Annen* (n 207) para 55. See: *Van Den Dungen* (n 22) s 2; *Van Schijndel* (n 30) s 1.

212 It would be useful, however—see: Aatifa Khan, ‘A “right not to be offended” under Article 10(2) ECHR? Concerns in the construction of the “rights of others”’ [2012] Eur Hum Rights Law Rev 191, 196 and 201.

213
*Dulgheriu* (n 5) [57] and [60]; Convention for the Protection of Human Rights and Fundamental Freedoms (European Convention on Human Rights, as amended) art 8. When one unpacks this right, it emerges that there are a number of interests at stake for clinic-users faced with demonstrations outside abortion clinics: personal autonomy, physical integrity, psychological integrity, and privacy.

214
*Annen* (n 207) para 55.

215
*Dulgheriu* (n 5) [91].

216 ibid [89].

217 ibid [89]–[95].

218 Grégor Puppinck, ‘Abortion and the European Convention on Human Rights’ (2013) 3 Irish J Legal Stud 142, 175 and 187.

